# Comparison of the Thermal and Mechanical Properties of Poly(phenylene sulfide) and Poly(phenylene sulfide)–Syndiotactic Polystyrene-Based Thermal Conductive Composites

**DOI:** 10.1021/acsomega.2c06152

**Published:** 2022-12-02

**Authors:** Yoldas Seki, Elif Kizilkan, Berkay Metin Leşkeri, Mehmet Sarikanat, Lutfiye Altay, Akin Isbilir

**Affiliations:** † Faculty of Science, Dokuz Eylul University, Buca, Izmir35160, Turkey; ‡ İzmir Eğitim Sağlık Sanayi Yatırım A.Ş., Turgutlu, Manisa45400, Turkey; § Mechanical Engineering Department, Ege University, Bornova, Izmir35040, Turkey

## Abstract

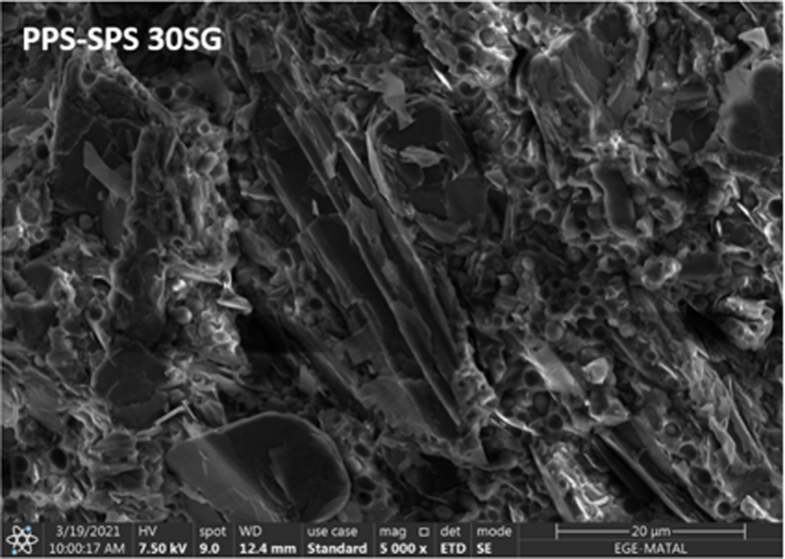

Syndiotactic polystyrene (SPS) has attracted considerable attention recently due to its high melting temperature, low cost, and relatively low density value. The aim of the study is to reveal whether a blend of PPS and SPS (PPS–SPS) can be used instead of PPS for high thermal stability, high mechanical performance, and high thermal conductive material applications. For this aim, poly(phenylene sulfide)/syndiotactic polystyrene-based carbon-loaded composite materials were prepared using a twin screw extruder. Two carbon-based materials, carbon fiber (CF) and synthetic graphite (SG), were used to improve the mechanical properties and thermal conductivity of the PPS–SPS blends. Through-plane conductivity values of PPS-30SG-10CF and PPS–SPS-30SG-10CF were obtained to be 13.67 and 12.92 W/mK, with densities of 1.55 and 1.50 g/cm3, respectively. It was demonstrated that PPS–SPS blend-based carbon-loaded composites have great potential to be used in thermal management applications with the advantages of relatively low cost and lightweight compared to PPS-based composites.

## 1 Introduction

The outstanding characteristics of conductive polymer composites make them widely popular in different applications such as heating elements, temperature-dependent sensors, self-limiting electrical heaters, switching devices, and antistatic materials for electromagnetic interferences and shielding of electronic devices.^1^ Among many fillers, graphite, abundantly available and easily functionalized to afford different applications, has unique properties such as high thermal and electrical conductivity, a low coefficient of thermal expansion, exceptional thermal resistance, a high thermal shock resistance, and relatively low cost.^1c^ The inherent fibrillar form of short carbon fibers (CF) has a higher tendency to form a three-dimensional network in the composites. Pramanik et al. indicated that the greater the surface-to-volume ratio of the carbon fiber, the more likely the interparticle contact, which will lead to higher conductivity and reduction in the percolation threshold.^1,2^ CF-reinforced composites offer considerable potential for mass reduction, especially in automotive applications. However, the raw material cost of CF is one of the main factors that limit its extensive use in this market.^3^ While graphite, cheaper than CF, may decrease the mechanical properties of polymers, CF-reinforced polymers can provide high strength and stiffness.^4^ Therefore, considering the material cost, the combination of synthetic graphite (SG) with a low amount of CF can provide both high thermal conductivity and high mechanical strength.

When the polymer matrix is expected to withstand high temperature and corrosive environments, high-temperature thermoplastics such as poly(phenylene sulfide) (PPS), poly(ether ether ketone) (PEEK), poly(ether sulfone) (PES), or even a liquid crystalline polymer (LCP) blended with the same polymer can be chosen.^5^ PPS is a semicrystalline aromatic polymer that demonstrates high thermal stability, outstanding chemical resistance, and excellent flame retardancy properties. However, due to the fact that PPS has poor thermal conductivity, the use of PPS in applications where thermal conductivity is required is limited.^6^ Moreover, glass fiber- or carbon fiber-reinforced PPS composites are also popularly utilized in the automobile, aerospace, and other widespread industrial sectors.^7^ However, the application of PPS is limited to a great extent due to its poor impact, toughness, and cost.^8^

According to the Thermal Conductive Additives Market Size, Share & Trends Analysis Report, the market shows an exponential increase in the demand for thermally conductive materials due to new applications such as electric vehicles, LED lighting assemblies, and complex automotive applications.^9^ Moreover, the use of lower-cost engineering resins such as nylons 6 and 66 and PC in thermal conductive compounds is gaining ground against higher-priced materials such as PPS, PSU, and PEI. However, the heat-proof property of the PC resin is much lower than that of the PPS resin.^10^ Besides, the heat distortion temperature of PA66 is low, and it easily absorbs water which deteriorates its mechanical properties and dimensional stability owing to the presence of amide groups in the molecular chain.^11^ Moreover, the polymer blends (PA66 and PPS) are immiscible on a molecular scale.^11^ PPS was also blended with PA46 to improve the tribological property of PPS, and blending decreased the density of PPS composites.^12^ However, the more polar PA46 absorbed more water than less polar PA6 and PA66 and, therefore, is more susceptible to moisture-induced dimensional growth.^13^ While improvement in materials is necessary to achieve weight savings without sacrificing performance, the property, manufacturability, and cost requirements for automotive structures are often not met by the existing set of advanced lightweight materials.^14^ However, it is stated that a 10% reduction in vehicle weight can result in a 6–8% fuel economy improvement.^14^ It is seen that without sacrificing the performance of the material, weight and price savings are demanded from the market.

Syndiotactic polystyrene (SPS), a new semicrystalline polymer, has attracted considerable attention because of its properties such as high melt temperature and high crystallinity.^15^ It is known that SPS has a lower cost nowadays and lower density than PPS–SPS and the PPS blend is partially compatible in the molten state. This study aims to reveal whether a blend of PPS–SPS having a lower density can be used instead of PPS for high thermal stability, high mechanical performance, and high thermal conductive composite materials. To improve the thermal conductivity and mechanical strength of the PPS–SPS blend, the combination of SG and CF was used.

## 2 Materials and Methods

### 2.1 Materials

PPS (25M88) and SPS (XAREC 90ZC) with density values of 1.355 and 1.04 kg/m^3^ were obtained from TORAY Industries, Inc. and IDEMITSU Chemicals, respectively. Carbon fiber (CF.OS.U5-6 mm) with a mean length of 6 ± 0.5 mm was purchased from Apply Carbon. Synthetic graphite (TIMREX KS44) with a density of 2.26 g/cm^3^ and a median particle size (*d*_50_) of 15.8 μm was supplied from IMERYS Graphite & Carbon (Switzerland).

### 2.2 Composite Manufacturing

CF and SG loaded PPS–SPS composites were melt-compounded using a corotating intermeshing twin screw extruder with 11 zones and an L/D of 48 (Leistritz 27 MAXX). In order not to degrade the mechanical properties of PPS considerably, PPS–SPS blends were prepared using 10 wt % SPS and 90 wt % PPS without any carbon loading. The SPS weight fraction was kept constant at 10 wt % in all composites containing SG and CF. The screw rotation speed was 500 rpm for all of the experiments, and the temperature profile of barrel sections from the hopper to die was set to 45-260-270-265-265-265-265-260-260-260-260-270 and 280 °C. After passing through the extruder die, the polymer strands entered the water bath and were cut into pellets using a pelletizer. Test specimens were obtained using pellets via the injection molding technique (Bole model BL90EK). PPS–SPS blends were molded at temperatures ranging from 300 to 280 °C. The mold temperature was about 120 °C. PPS–SPS blends were able to work at lower injection temperatures compared to PPS, which was molded at temperatures ranging from 320 to 300 °C.

### 2.3 Characterization Methods

#### 2.3.1 Density

The density of the specimens was determined using an electronic densimeter (density balance), MD-200S, in accordance with the ISO1183-1 standard.^16^ The density of each composite was measured by calculating the average density of three specimens.

#### 2.3.2 Thermogravimetric Analysis (TGA)

Thermogravimetric analyses of PPS, PPS–SPS, and their composites were performed using a TG analyzer (TA Instruments Inc., TGA-Q50). The analyses were performed at a heating rate of 10 °C/min in the temperature range of 30–900 °C under a nitrogen atmosphere.

#### 2.3.3 Differential Scanning Calorimeter (DSC) Analysis

Differential scanning calorimeter analyses were conducted using a DSC Q20 (TA Instruments Inc., DSC Q20). The samples were heated from 10 to 300 °C at a rate of 10 °C/min under a nitrogen atmosphere. After an isothermal scan for 3 min, the temperature decreased to −80 °C at the same cooling rate. In the last stage, it was heated from −80 to 300 °C with a heating rate of 10 °C/min.

#### 2.3.4 Thermal Conductivity Measurement

The thermal diffusivity (α) values were measured using the Discovery Xenon Flash DXF 200 (TA Instruments) at room temperature. The Discovery Xenon Flash DXF 200 employs a high-speed xenon pulse delivery system that provides a high degree of accuracy for measuring thermal diffusivity ranging from 0.01 to 1000 mm^2^/s, according to the ASTM 1461-07 standard.^17^ Sample sizes were 2.54 cm and 0.5 mm in diameter and thickness, respectively. The density (ρ) and the specific heat capacity (*C*_p_) of the samples were measured using the Densimeter MD-200S and DSC Q20 (TA Instruments), respectively. Then, in-plane and through-plane thermal conductivity values of PPS and PPS–SPS-based composites (κ) were calculated according to eq 1.^18^
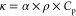
1

#### 2.3.5 Heat Distortion Temperature (HDT) Testing

HDT values of PPS, PPS–SPS, and their composites were obtained according to the ISO 75 standard under a specific load of 1.8 MPa.^19^

#### 2.3.6 Mechanical Testing

The tensile properties of PPS and PPS–SPS-based composites were measured with a Hegewald & Peschke Inspect 20 universal testing machine equipped with a video extensometer system (Hegewald & Peschke Inspect 20 Noncontact Video Extensometer) at a crosshead speed of 50 mm/min according to the ISO 527 standard.^20^ The flexural properties of PPS and PPS–SPS-based composites were determined with a 2 mm/min deformation rate according to the ISO 178 standard.^21^ The Izod impact strength and Charpy impact strength values of PPS and PPS–SPS-based composites were measured according to ISO 180 and 179 standards, respectively.^22^ Unnotched impact strengths of the samples having dimensions of 80 mm × 10 mm × 4 mm were also determined. The average of tests was recorded for tensile, flexural, and impact properties.

#### 2.3.7 Scanning Electron Microscopy (SEM) Analysis

The fracture surfaces of tensile test specimens were examined by a scanning electron microscope (Thermo Scientific Apreo S) operated at 7.5 kV. Gold was deposited on the surface of the specimens using a plasma sputtering apparatus.

## 3 Results and Discussion

### 3.1 Density Results

The density values of PPS, PPS–SPS blend, and their composites can be seen in Table 1. The density values of SG- and/or CF-loaded composites are higher than those of the PPS and PPS–SPS blend. Although the density of PPS was measured as 1.35, 1.30 g/cm^3^ was obtained when 10 wt % SPS was used in the PPS–SPS blend. From Table 1, it is seen that the density of the composites increased with increasing SG and CF weight fractions. This is due to the fact that the density values of carbon fiber (1.8 g/cm^3^)^23^ and synthetic graphite (2.26 g/cm^3^) are higher than the PPS and PPS–SPS blend.

**Table 1:** Density values of samples

Sample	Density (g/cm^3^)
**PPS**	1.35 ± 0.04
**PPS–SPS**	1.30 ± 0.03
**PPS–SPS-10SG**	1.35 ± 0.05
**PPS–SPS-20SG**	1.38 ± 0.03
**PPS–SPS-30SG**	1.46 ± 0.06
**PPS-30SG**	1.51 ± 0.02
**PPS-30SG-5CF**	1.54 ± 0.04
**PPS–SPS-30SG-5CF**	1.48 ± 0.03
**PPS-30SG-10CF**	1.55 ± 0.02
**PPS–SPS-30SG-10CF**	1.50 ± 0.04

### 3.2 TGA Results

**Figure 1:**
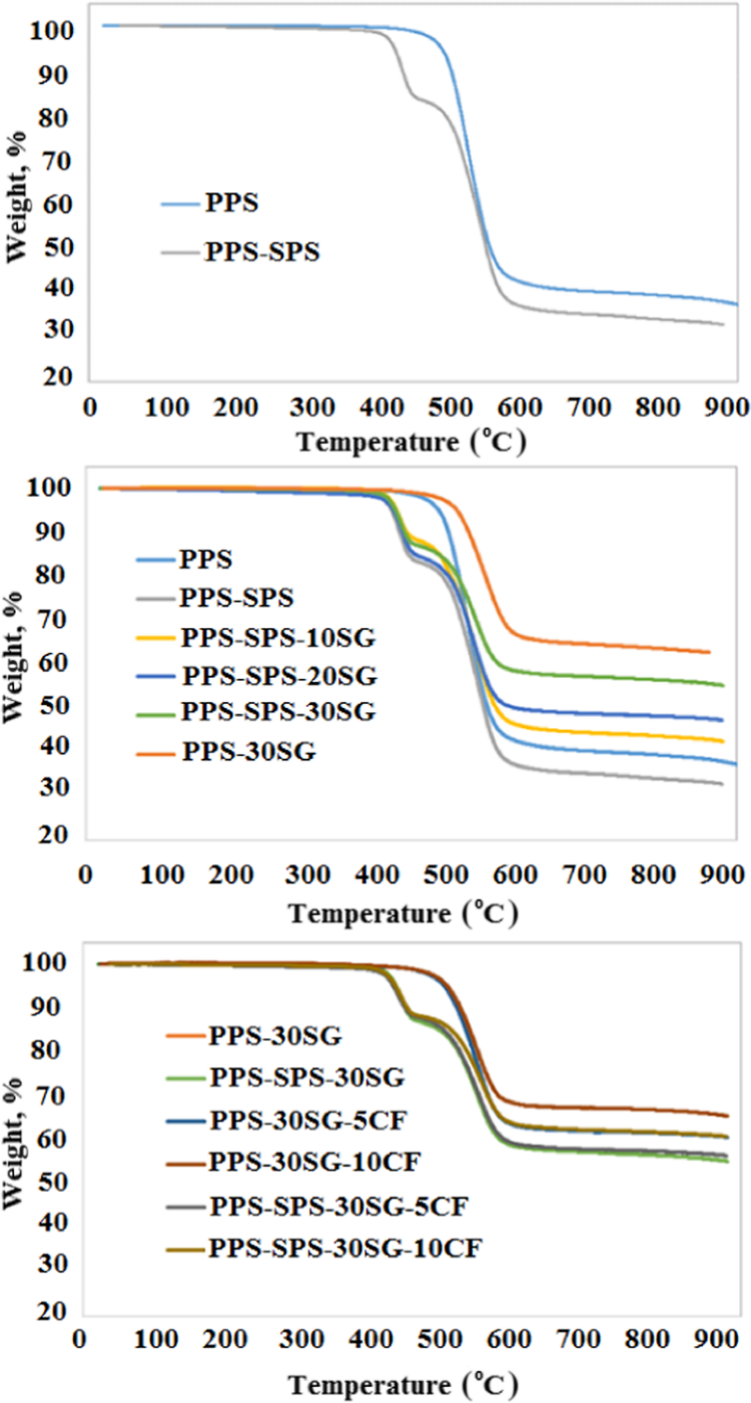
TGA thermograms of the PPS, PPS–SPS blend, and their composites.

Thermogravimetric analyses of PPS and PPS–SPS, synthetic graphite, and carbon fiber-loaded PPS–SPS composites with different weight fractions are shown in Figure 1. In the literature, the decomposition temperature of the raw PPS material is 400–700 °C.^24^ In this study, the maximum decomposition temperature of raw PPS was determined as 526 °C. Table 2 shows the varying maximum decomposition temperatures depending on the polymer, filler, and reinforcement materials used in different weight fractions. As can be seen from Figure 1, the PPS–SPS blend has two degradation steps. The first degradation step takes place due to SPS degradation because of the relatively lower thermal stability of SPS compared to PPS. It can be said that SPS blending of PPS has led to poor heat resistance. It was also observed that a higher maximum decomposition temperature was obtained for PPS (546 °C) in the PPS–SPS blend compared to raw PPS (526 °C). The weight loss value of PPS was obtained to be about 60%. As the proportion of the raw PPS material in the PPS–SPS blend decreases to 90%, the amount of weight loss in the blend increases due to the degradation of SPS completely in the studied temperature range. In the PPS–SPS–SG composite seen in Table 2, a slight change was observed for the maximum decomposition temperatures due to the increasing SG weight fraction. As can be seen from Figure 1, the decrease in weight loss is due to the increasing SG and CF weight fractions. The maximum degradation temperatures of PPS and PPS-30SG were obtained to be 526 and 553 °C, respectively. It can be said that the thermal stability of PPS was improved by adding SG, which is compatible with the result obtained using poly(ethylene terephthalate) and graphite.^25^ From Table 2, it can also be added that the maximum decomposition temperatures of PPS in both PPS and SPS-based composites are higher than that of raw PPS. In the PPS–SPS–SG-CF composite, the highest maximum decomposition temperature was obtained using a larger weight fraction of CF. However, for PPS–SG-CF composites, the CF loading decreased the maximum decomposition temperature of PPS-30SG.

**Table 2:** TGA results of PPS and PPS–SPS with various synthetic graphite (SG) and carbon fiber (CF) weight fractions

Sample	Max. degradation temperature (°C)	Weight loss (%)
**PPS**	526	60.3
**PPS–SPS**	433 and 546	66.9
**PPS–SPS-10SG**	435 and 540	55.9
**PPS–SPS-20SG**	438 and 537	51.5
**PPS–SPS-30SG**	437 and 540	44.6
**PPS-30SG**	553	36.5
**PPS–SPS-30SG-5CF**	433 and 537	43.4
**PPS-30SG-5CF**	535	39.2
**PPS–SPS-30SG-10CF**	436 and 545	37.1
**PPS-30SG-10CF**	538	34.4

### 3.3 DSC Results

**Figure 2:**
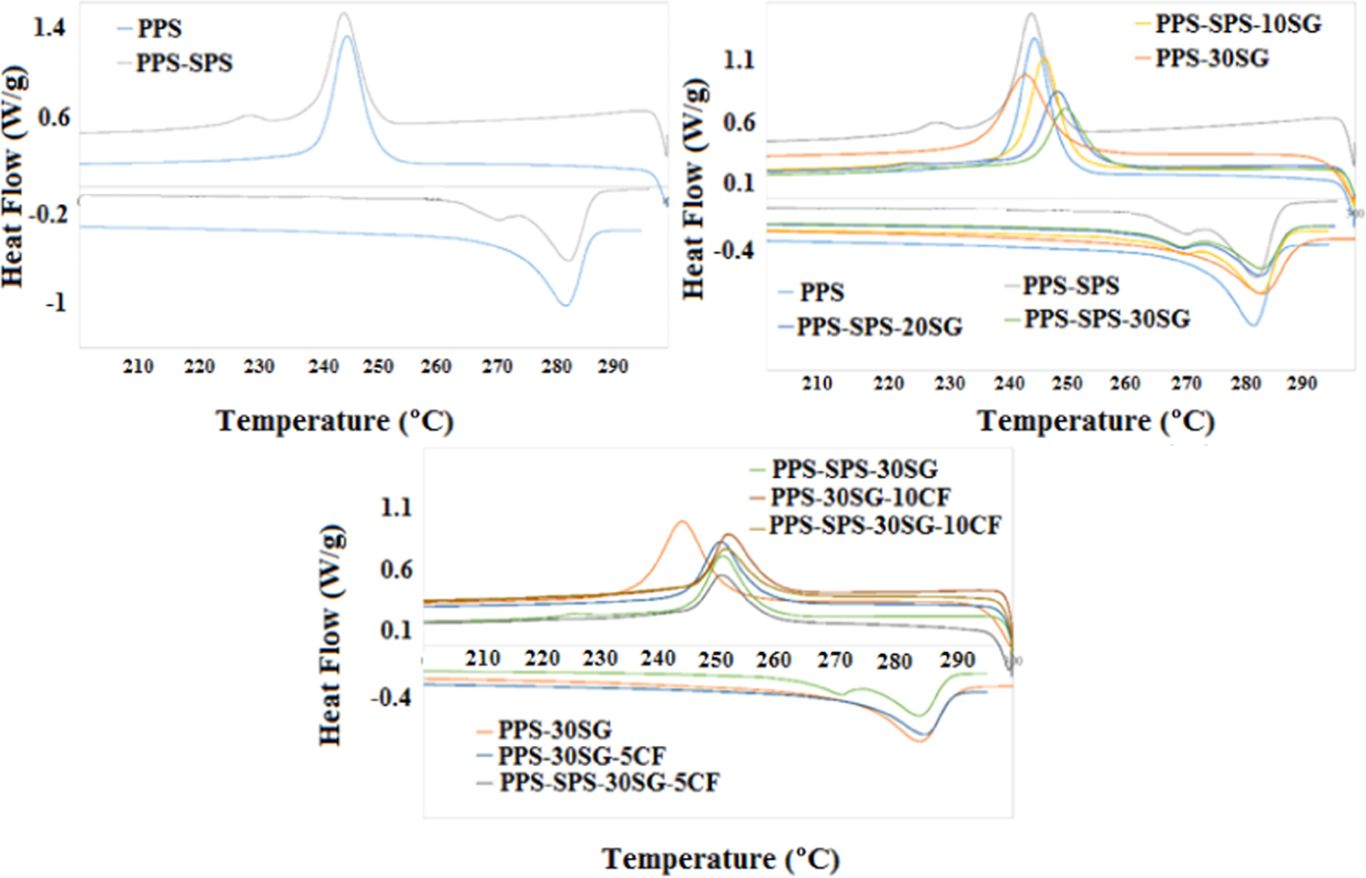
DSC curves of PPS, the PPS–SPS blend, and their composites.

The effect of synthetic graphite and carbon fiber on the melting and crystallization behaviors of PPS and PPS–SPS is shown in Figure 2. DSC results obtained from Figure 2 are summarized in Table 3. As can be seen from the DSC curves of the PPS and PPS–SPS blend, a small melting peak lower than 284 °C, the melting temperature of PPS, was observed. This peak was observed due to the melting behavior of SPS in the PPS–SPS blend in spite of the presence of SPS at a lower amount (10 wt %). The melting temperature of PPS–SPS did not change significantly with the addition of filler and/or reinforcement. However, crystallization temperatures increased with the addition of synthetic graphite into PPS–SPS composites. There was no significant change in the crystallization temperature of carbon fiber-reinforced PPS–SPS–SG composites. On the other hand, there was an increase in the crystallization temperatures of the graphite and carbon fiber-loaded composite groups compared to PPS and PPS–SPS. The crystallization temperatures of PPS-30SG, PPS-30SG-5CF, and PPS-30SG-10CF were obtained to be 244, 257, and 259 °C, respectively, which clearly shows the increases in the crystallization temperatures due to adding CF into the PPS matrix. It may be an advantage for the injection molding technique to reduce the cycle time and for thermoforming to increase the throughput. The melting enthalpy (Δ*H*_m_) and crystallization enthalpy (Δ*H*_c_) decreased with the addition of SG and CF. The decrease in the melting enthalpy indicates that less energy was required to melt the composites.^26^ Moreover, the decrease in Δ*H*_c_ can be explained by the fact that the weight fraction of the polymer decreased with the addition of SG and CF.

**Table 3:** DSC results of PPS and PPS–SPS with various synthetic graphite and carbon fiber weight fractions

Sample	*T*_m_ (°C)	Δ*H*_m_ (J/g)	*T*_c_ (°C)	Δ*H*_c_ (J/g)
**PPS**	283	40.7	246	46.4
**PPS–SPS**	284	34.2	245	45.0
**PPS–SPS-10SG**	284	34.3	252	34.3
**PPS–SPS-20SG**	284	31.5	249	28.9
**PPS–SPS-30SG**	284	28.5	251	23.7
**PPS-30SG**	284	18.2	244	21.6
**PPS-30SG-5CF**	283	22.4	257	27.0
**PPS–SPS-30SG-5CF**	285	19.2	258	21.9
**PPS-30SG-10CF**	285	17.3	259	26.6
**PPS–SPS-30SG-10CF**	285	23.1	259	26.6

### 3.4 Thermal Conductivity

**Figure 3:**
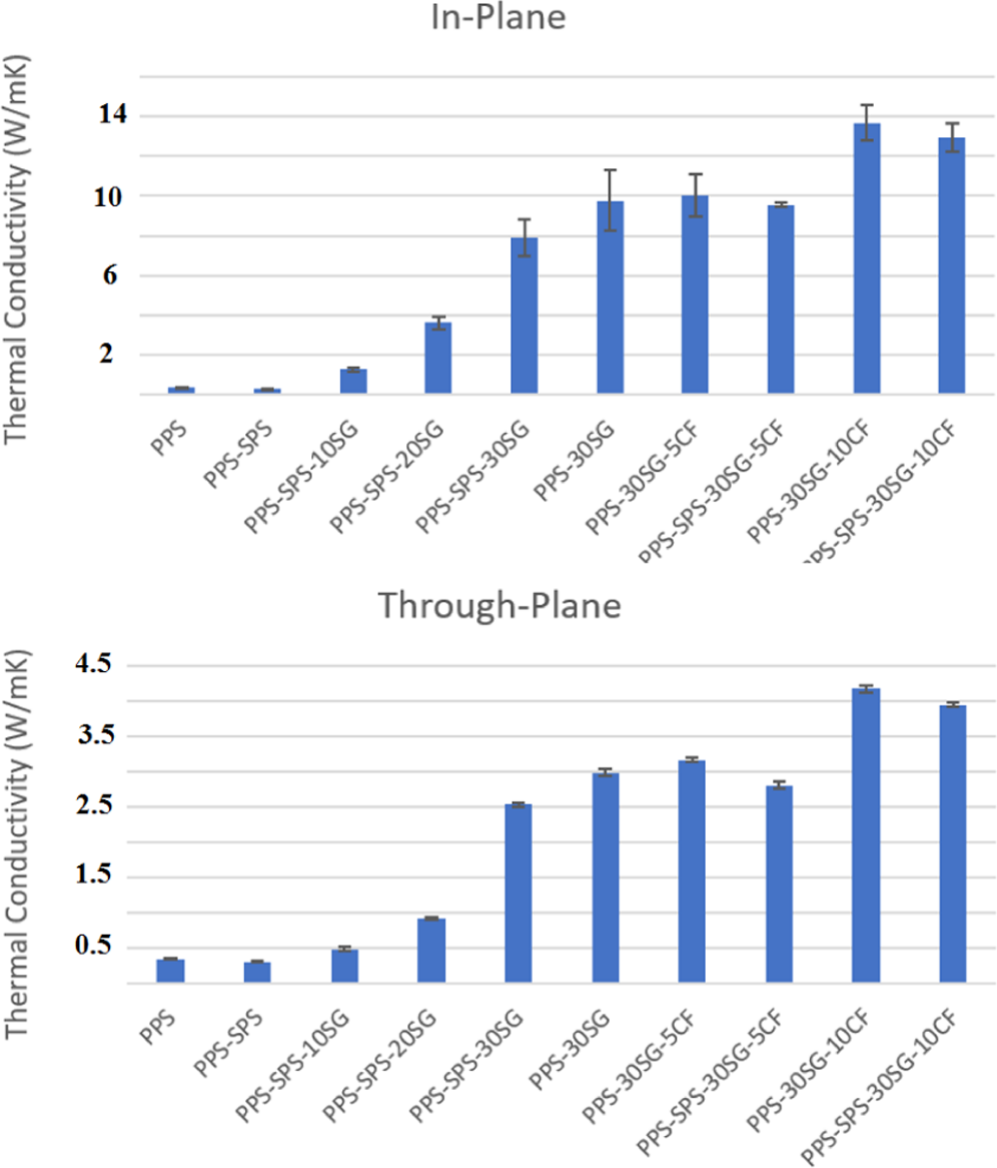
Thermal conductivity values of SG and CF-loaded PPS and PPS–SPS-based composites.

In-plane and through-plane thermal conductivity values are given in Figure 3. It has been shown that thermal conductivities increased in both directions, as expected with the increasing weight fraction of carbon loadings, independent of SG or CF. The addition of SPS decreased the thermal conductivity value of PPS from 0.35 to 0.31 W/mK. The incorporation of 30 wt % SG into PPS increased the thermal conductivity values to 9.76 and 2.98 W/mK for in-plane and through-plane, respectively. However, when 30 wt % SG was used in the PPS–SPS blend, a slight decrease was observed in the thermal conductivity values, as expected in composites, due to the lower thermal conductivity value of the PPS–SPS blend than that of PPS.

As seen in Figure 3, SG causes further improvement in the in-plane thermal conductivity due to the layered nature of SG that leads to the parallel alignment of SG particles within the composites. In-plane thermal conductivity values were obtained to be 1.28, 3.64, and 7.91 W/mK for 10, 20, and 30 wt % SG-added PPS–SPS-based composites, respectively. In the case of carbon fiber and synthetic graphite loadings, both in-plane and through-plane thermal conductivity values increased. However, one can note that the CF loading into the PPS–SPS blend resulted in higher through-plane thermal conductivity values due to the high aspect ratio of the carbon fibers, which may be the reason for a better thermal conductivity network between SG and CF. This result is foreseeable because CFs with high aspect ratios show a highly anisotropic thermal conductivity behavior. In other words, the thermal conductivity value of CF is much higher in the longitudinal direction than in its transverse direction.^27^ The largest thermal conductivity values in this study were obtained to be 4.17 W/mK (through-plane) and 13.67 W/mK (in-plane) for the PPS-30SG-10CF composite. Comparatively, PPS–SPS blend-based composites with 30 wt % SG and 10 wt % CF presented 12.92 and 3.94 W/mK in-plane and through-plane thermal conductivities, respectively. This result shows that PPS–SPS blend-based carbon-loaded composites demonstrated good potential for use in thermal management applications as an economical alternative to PPS-based composites.

### 3.5 HDT Results

HDT values of PPS, PPS–SPS, and their composites are presented in Table 4. Addition of 10 wt % SPS into PPS decreased the HDT value of PPS and 10 wt % SG loading into PPS–SPS decreased the HDT value of PPS. However, SG loading of 30 wt % and CF loadings at all weight fractions (5–10 wt %) into PPS–SPS increased the HDT value of PPS–SPS. It is known that HDT is used to determine elevated temperature performance in plastic materials and is often industrially utilized in the material selection process as the maximum continuous use temperature.^28^ HDT values of PPS–SPS and its composites are presented in Table 4. 10 wt% SPS adding into PPS decreased the HDT value of PPS by 6 °C. However, it is possible to increase the HDT value of the PPS–SPS blend by SG loadings. When HDT values of PPS-30SG-5CF and PPS–SPS-30SG-5CF were compared, PPS-30SG-5CF had a higher HDT value than that of PPS–SPS-30SG-5CF. Besides, PPS-30SG-10CF has the highest HDT value in this study. It is seen that the PPS-based composite has a higher continuous use temperature than the composite of the PPS–SPS blend. The HDT value of PPS–SPS-30SG-5CF is higher than that of PPS–SPS-30SG-10CF because of the fact that PPS–SPS-30SG-10CF probably has a poor distribution of CF within the matrix. Considering the thermal analyses, it can be said that the disadvantage of PPS–SPS over PPS is thermal stability. The maximum degradation of PPS–SPS-30SG-10CF is lower than that of PPS-30SG-10CF due to the lower decomposition temperature of SPS. The second disadvantage of blending SPS with PPS is poor heat resistance. The HDT value of PPS-30SG-10CF is 40 °C higher than that of PPS–SPS-30SG-10CF. Therefore, the PPS-based composite has a higher continuous use temperature than the composite of the PPS–SPS blend.

**Table 4:** HDT values of samples

Sample	HDT (A) °C
**PPS**	84
**PPS–SPS**	78
**PPS–SPS-10SG**	101
**PPS–SPS-20SG**	109
**PPS–SPS-30SG**	114
**PPS-30SG**	121
**PPS-30SG-5CF**	240
**PPS–SPS-30SG-5CF**	231
**PPS-30SG-10CF**	240
**PPS–SPS-30SG-10CF**	200

### 3.6 Mechanical Properties

The tensile and flexural properties of PPS and PPS-based composites are given in Table 5. Except for PPS–SPS-20SG, the SG loading into the PPS–SPS blend has not led to a considerable decrease in the tensile strength. However, 20 and 30 wt % SG loadings into the PPS–SPS blend decreased the flexural strength of the PPS–SPS blend by 7 and 9%, respectively. 5 wt % CF loading into the PPS–SPS blend containing 30 wt % SG increased the tensile and flexural strength values by 69 and 18%, respectively. Besides 10 wt % CF loading has led to 112 and 38% increments in tensile and flexural strength values, respectively. Comparing the tensile and flexural strength values of PPS–SPS-30SG-5CF and PPS-30SG-5CF, the tensile strength and flexural strength of PPS–SPS-30SG-5CF are about 13 and 8% lower than those of PPS-30SG-5CF, respectively. Moreover, the tensile strength and flexural strength values of PPS–SPS-30SG-10CF are greater than those of PPS-30SG-10CF, which may be due to poor dispersion of CF within the PPS. It is also seen that the CF loading into PPS and the PPS–SPS blend resulted in larger flexural modulus values. It is known that the addition of rigid particles to a thermoplastic matrix leads to an increase in modulus values.^29^ The elongation at break values of the PPS–SPS blend and its composites are lower than 1.5%.

**Table 5:** Tensile and flexural properties of samples

Sample	Tensile strength (MPa)	Elongation at break (%)	Flexural strength (MPA)	lFlexural modulus (MPa)
**PPS–SPS**	51.0 ± 1.3	1.2 ± 0.2	102 ± 3	3044 + 103
**PPS–SPS-10SG**	48.9 ± 3.9	1.5 ± 0.2	108 ± 5	7327 ± 189
**PPS–SPS-20SG**	37.2 ± 3.1	<1	95 ± 2	6013 ± 13
**PPS–SPS-30SG**	53.7 ± 2.4	<1	93 ± 1	7566 ± 26
**PPS-30SG**	56.0 ± 7.0	<1	96 ± 4	8563 ± 573
**PPS-30SG-5CF**	98.7 ± 2.2	<1	130 ± 3	11420 ± 49
**PPS-30SG-10CF**	70.3 ± 6.1	<1	157 ± 6	16692 ± 541
**PPS–SPS-30SG-5CF**	85.5 ± 8.4	<1	120 ± 3	12652 ± 384
**PPS–SPS-30SG-10CF**	107.7 ± 4.1	<1	141 ± 2	15163 ± 405

**Figure 4:**
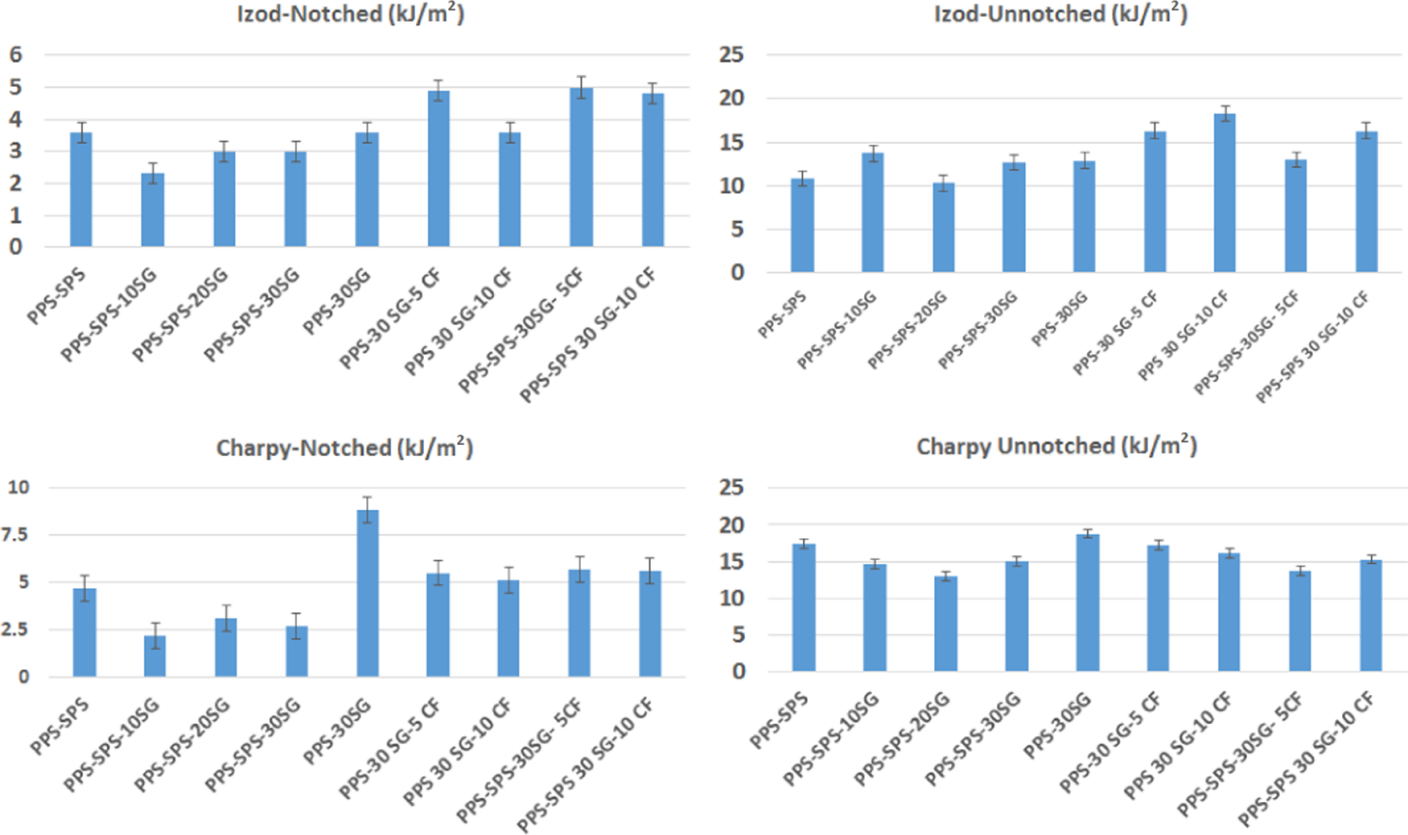
Izod impact strength and Charpy impact strength values of samples.

The Izod notched/unnotched impact strength and Charpy notched/unnotched impact strength values of PPS and PPS–SPS-based composites are presented in Figure 4. The Izod notched impact strength and Charpy notched impact strength values of the PPS–SPS blend decreased with the SG loading. This reduction may be attributed to the rigid graphite filler particles that cannot be deformed by external stress in the specimen. However, these particles act only as stress concentrators or crack initiation sites during the deformation process.^29,30^ As the SG weight fraction increases, agglomeration increases and the interfacial adhesion weakens, which induces microspaces between SG and the polymer matrix. This may lead to numerous microcracks, and rapid crack propagation through the material takes place.^29^

However, the CF loading has resulted in higher Izod notched impact strength and Charpy notched impact strength values. The SG loading into the PPS–SPS blend has led to a larger decrease in the Charpy notched impact strength. The Izod unnotched impact strength and Charpy unnotched impact strength values of PPS-30SG-5CF and PPS-30SG-10CF are greater than those of PPS–SPS-30SG-5CF and PPS–SPS-30SG-10CF. However, the Izod notched and Charpy notched impact strengths of PPS–SPS-30SG-10CF are about 33 and 10% higher than those of PPS-30SG-10CF. This may be due to the fact that SPS particles dispersed in composites acted as stress concentration points when subjected to external impact loading. This creates crazes and shear bands in the matrix and consumes more energy.^31^

### 3.9 SEM Analysis

**Figure 5:**
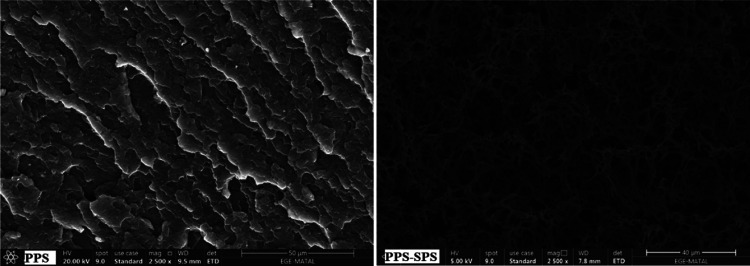
SEM images of PPS and the PPS–SPS blend.

SEM microstructures of PPS and the PPS–SPS (90/10) blend are presented in Figure 5. The immiscibility of the PPS–SPS blend can be seen in Figure 5.

**Figure 6:**
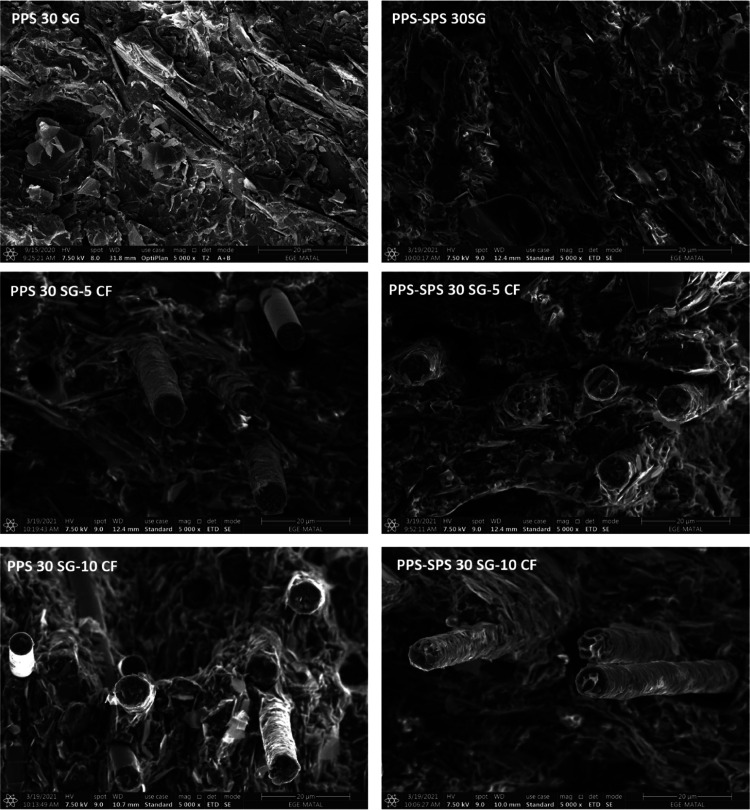
SEM images of SG- and CF-loaded PPS and PPS–SPS-based composites.

The same result was also obtained by Hwang et al., 2002.^32^ From the SEM image of the PPS–SPS blend, it is seen that the particle size of SPS is in the range of 1–5 μm. The boundaries of SPS particles are easily noticeable and are separated from the PPS matrix. Moreover, many voids stemming from the detachment of the SPS particles are seen in the SEM image of the PPS–SPS blend, which indicates a poor adhesion at the interface between the domain and the PPS matrix. The presence of SPS and the porous structure of the PPS–SPS blend in the SEM image of the PPS–SPS blend decreases the density of the PPS–SPS blend. The fracture surfaces of synthetic graphite and/or carbon fiber-loaded PPS and PPS–SPS blends are shown in Figure 6. The lamellar structure of SG is clearly visible in all composites, especially in PPS-30SG and PPS–SPS-30SG. In the case of the hybrid carbon loading, SEM images demonstrate that carbon fibers are embedded in the polymer matrix; furthermore, they are not separated from the matrix. The absences of circular holes or detached fibers are in good agreement with the mechanical results. When SEM images are examined, it is seen that both SG and CF are homogeneously distributed in the composites. It is also shown that as the weight fraction of CF increases from 5 to 10%, more CF is observed, as expected. The spherical particles observed in all PPS–SPS blend samples with dimensions of 2–3 μm represent the minor component of PPS/SPS mixtures, in other words, the SPS matrix. Many spherical voids seen in SEM images of composites with the PPS–SPS blend could be due to the detachment of the particles during the fracture process. Moreover, the difference between the melt viscosity of the two homopolymers (PPS and SPS) may result in immiscibility due to the high interfacial tension between components during the melt mixing process in the extruder.^32^ From Figure 5, SG seems to have poor compatibility with the matrix since an obvious interface is observed. It is known that good compatibility between SG and the polymer is the key to achieving good dispersion of SG and better properties of the composite.

## 4 Conclusions

This paper deals with the effect of the carbon fiber and/or synthetic graphite loadings into PPS and PPS–SPS blends on the mechanical and thermal properties of composite materials. In-plane and through-plane thermal conductivity values increased with the increasing weight fraction of carbon loadings, independent of SG or CF. The largest thermal conductivity values in this study were obtained as 13.67 and 4.17 W/mK in through-plane and in-plane directions for PPS-30SG-10CF composites, respectively. When the PPS–SPS blend was used in composites containing 30 wt % SG and 10 wt % CF, 12.92 and 3.94 W/mK were obtained for through-plane and in-plane thermal conductivities, respectively. However, the density values of PPS-30SG-10CF and PPS–SPS-30SG-10CF were measured to be 1.55 and 1.50 g/cm^3^, respectively. Although the PPS–SPS blends have advantages such as low density and cost over PPS, considering the thermal conductivity, impact values, and the mechanical and thermal properties of PPS-based composites (PPS–SG and PPS-CF-SG) and PPS–SPS-based composites (PPS–SPS–SG and PPS–SPS–SG-CF), PPS-based composites exhibit better in-plane and through-plane conductivities, mechanical properties (impact, tensile strength, and flexural strength values), and thermal stability. This may be due to the partially compatible nature of PPS and SPS. However, these differences are comparatively close to each other. This study imparts the role of SPS in the PPS–SPS blends as a lightweight and cost-effective material without degrading other properties so much.
